# Biological pretreatment and fermentation of *Panicum antidotale* biomass for pectinase production by *Bacillus vallismortis*

**DOI:** 10.1371/journal.pone.0339181

**Published:** 2026-01-23

**Authors:** Amal Siraj, Uroosa Ejaz, Masooma Hassan, Mohammed Alorabi, Abdullah K. Alanazi, Muhammad Sohail

**Affiliations:** 1 Department of Microbiology, University of Karachi, Karachi, Pakistan; 2 Department of Applied Sciences, Hamdard University, Karachi, Pakistan; 3 Department of Biosciences, Faculty of Life Science, SZABIST University, Karachi, Pakistan; 4 Department of Biotechnology, College of Sciences, Taif University, Taif, Saudi Arabia; 5 Department of Chemistry, College of Science, Taif University, Taif, Saudi Arabia; ENCB-IPN: Instituto Politecnico Nacional Escuela Nacional de Ciencias Biologicas, MEXICO

## Abstract

Halophytic plants are renewable source of lignocellulosic biomass, however, are underexplored for their utilization as a source of fermentation raw material. Similar to glycophytes, biomass from halophytes contains lignin that is removed to provide access to the fermentable carbohydrates. This study was designed to investigate biomass of a halophytic plant, *Panicum antidotale,* for pectinase production from a halotolerant strain of *Bacillus vallismortis* MH 10. Laccase from a fungus, *Trametes pubescens* MB 89 was employed as a pretreatment agent to remove lignin. The pectin content of *P. antidotale* biomass was also determined and compared with the pectin extracted from orange peels (OP). The changes in the *P. antidotale* biomass and pectin were investigated using Fourier Transform Infrared spectroscopy (FTIR) and Scanning Electron microscope (SEM). The strain MH 10 produced 17.39 IU mL^-1^ pectinase in the medium containing *P. antidotale* biomass supplemented with fungal laccase. The data showed that *P. antidotale* biomass has a meagre quantity of pectin (2.8%) compared to OP (14.9%). Yet the strain MH 10 produced 315 IU g^-1^ pectinase by fermenting pectin from *P. antidotale* but only 76.32 IU g^-1^ pectinase was obtained by using OP as substrate which showed preference of this strain towards halophytic substrate. The analysis further revealed that the strain effectively utilized 94% pectin content of *P. antidotale* biomass. The FTIR spectra corresponded to the changes in pectin spectrum indicating pectin consumption from *P. antidotale* biomass. The SEM images confirmed the laccase-mediated porosity and fragility in the biomass. Hence, this study provides a novel utilization of biomass from halophytes as a chemical feedstock.

## 1. Introduction

Lignocellulosic biomass is the most abundant and promising resource derived from plants for processing into useful products. It is primarily composed of cellulose, hemicellulose, and lignin [1]. It is a sustainable alternative to fossil fuels for manufacturing industrial goods [2]. The most frequently reported sources of lignocellulosic biomass include sugarcane bagasse [3], corn husks [4], and fruit peels [5]. In addition to the residues from food-based crops, the biomass from halophytes has been identified as a promising chemical resource for generating greener bio-products [6,7].

The halophytes grow in saline soil and, therefore, they do not compete with typical food crops for land and water resources [8,9]. Halophytes have evolved several strategies to withstand salinity land, such as by storing salt in their tissues [10] and producing secondary metabolites [11]. The lignocellulosic biomass from halophytes is a great resource for producing value added products including second generation of bioethanol [8], biodiesel [12], high quality light weight paper, fiber reinforced polymer [13], and pectinolytic enzymes [14]. Pakistan hosts ~410 halophytic species as reported by Khan & Qaiser, [15]. Notable halophytes found in the coastal areas of Sindh province include *Suaeda fruticosa, Heliotropium curassavicum, Haloxylon stocksii* [16], *Phargmites karka, Panicum antidotale, Halopyrum mucronatum*, and *Desmostachya bipinnata* [7].

Halophytes-derived biomass like other plant-based materials requires an essential pretreatment step before saccharification to improve the product yield [17]. The choice of pretreatment is determined by parameters such as cost effectiveness, biomass digestibility, and the release of non-toxic chemicals [18].

As a biological pretreatment agent, laccase has been recognized for its broad substrate specificity to catalyze both phenolic and non-phenolic substrates [19,20], which ultimately enhances subsequent saccharification of biomass [21]. Unlike chemical pretreatment, laccase-mediated pretreatment does not release harmful by-products [1] and preserves polysaccharide constituents (including cellulose, xylan, and pectin) of the biomass [22].

Literature search suggests that halophyte-derived biomass has been largely exploited for its cellulose and hemicellulose contents. However, reports on the utilization of pectic materials from such biomass are scarce. This is the first report describing the pectin content in *P. antidotale* biomass and its utilization for pectinase production by halotolerant bacterium, *B. vallismortis* MH10. We employed laccase from *T. pubescens* MB 89 as a pretreatment agent to render the process environmentally friendly. Pectin was extracted from *P. antidotale* and compared with the pectin extracted from orange peels (OP) for pectinase production. Furthermore, structural and morphological analysis of the substrate were performed by using FTIR and SEM.

## 2. Methodology

### 2.1 Microbial strains and inoculum preparation

Bacterial strains MH 1 and MH 10 of *B. vallismortis* and fungal strain MB89 of *T. pubescens* were obtained from the culture collection of the Department of Microbiology, University of Karachi. *B. vallismortis* MH 1 was previously reported for its cellulase, xylanase, and pectinase production [7], whereas, MH 10 was reported for pectinase production [14]. The fungal strain MB89 was reported to produce laccase in sugarcane bagasse-containing medium [23]. In this study, a single colony of MH 1 and MH 10 was separately transferred to nutrient broth for inoculum preparation (Oxoid, USA) and incubated at 37°C and 50ºC, respectively, for 24 h. The inoculum density was maintained at 0.6 OD_600_. The inoculum of *T. pubescens* MB89 was prepared by transferring a loopful of spore suspension from the stock culture to Sabouraud’s Dextrose agar (Oxoid, USA) plate and incubating at 25°C for 7 days. An agar piece (~1 cm^2^) covered with fungal spores was used as an inoculum in subsequent experiments.

### 2.2 Collection of halophytic plants

Five halophytic plants, *Desmostachya bipinnata* (DB), *Halopyrum mucronatum* (HM), *Salvadora persica* (SP), *Panicum antidotale* (PA), and *Phragmites karka* (PK) were obtained from the Institute of Sustainable Halophyte Utilization, University of Karachi. The plants were identified by comparing with the already identified plants present at the Herbarium of University of Karachi, Pakistan.

### 2.3 Production of cellulase

Mineral salt medium supplemented with carboxymethyl cellulose (MSM-CMC) was used for cellulase production, as described earlier [24]. Briefly, an inoculum of 10% MH 1 was transferred to MSM-CMC and incubated at 37°C for 24 h. After incubation, the cell-free culture supernatant was obtained by centrifugation at 2500 *x g* for 15 min and used as crude cellulase preparation.

### 2.4 Cellulase assay

Carboxymethyl cellulose (CMC; 0.5% w/v) in sodium citrate buffer (50 mM, pH 4.8) was used as a substrate to perform cellulase assay. Cellulase preparation from MH 1 (25 μL) was mixed with 25 μL of the substrate prepared in 50 mM sodium citrate buffer (4.8 pH) and incubated for 15 min at 37°C [7]. The amount of reducing sugars released by the enzyme was determined by dinitrosalicylic acid method [25].

### 2.5 Production of Laccase

Solid state fermentation (SSF) of sugarcane bagasse was carried out using 2 g of sugarcane bagasse in a 250 mL Erlenmeyer flask as reported by Abbas et al. [23] with slight modifications. An inoculum of a 1 cm^2^ block of *T. pubescens* MB89 was taken from SDA plate and transferred to sugarcane bagasse. Moisture content was maintained at 80% by adding distilled water and the flasks were incubated at 25°C for 10 days. After incubation, 25 mL of 50 mM sodium citrate buffer (pH 3) containing 0.05% w/v Tween 80 was added, and the mixture was placed in a shaking incubator at 25°C for 2 h to extract laccase. The slurry was then filtered using a muslin cloth and centrifuged at 2500 *× g* for 15 min. The resulting cell-free supernatant was collected as laccase preparation.

### 2.6 Laccase assay

Laccase activity was determined using the substrate 2,2-azino-bis 3-ethylbenzothiazoline-6-sulfonic acid (ABTS). From a 20 mM stock of ABTS, 5 mM of working solution was prepared in 50 mM sodium citrate buffer (pH 3). The laccase preparation (10 µL) was mixed with 100 µL of the working solution and 890 µL of 50 mM sodium citrate buffer (pH 3). The difference in the OD_420_ was measured against 50 mM sodium citrate buffer (pH 3) and units of laccase were calculated as reported by Abbas et al. [23].

### 2.7 Pretreatment of halophytic plants

Laccase and cellulase were employed for pretreatment and saccharification of halophytic plant biomass, respectively under the conditions reported earlier [24]. The cellulase from strain MH 1 and laccase from *T. pubescens* MB 89 were standardized at 40 IU g^-1^ substrate. The substrates were added to 50 mM sodium citrate buffer containing 0.2% (w/v) sodium azide. The reaction was carried out with different modifications for 24 h, adjusting different pH and temperatures as mentioned in Table 1. Aliquots were collected from the reaction mixture after 0 and 24 h to estimate the release of reducing sugars using the DNS method [25].

**Table 1:** Experimental conditions for enzymatic pretreatment of halophytic biomass using laccase (*T. pubescens* MB 89) and cellulase (*B. vallismortis* MH 1). Different treatments were carried out under varying pH and temperature to evaluate reducing sugar release after 24 h of reaction.

Treatment	Enzyme	pH	Temperature (ºC)
i.	Laccase pretreatment	3	30
ii.	Laccase pretreated biomass further treated with cellulase	5	37
iii.	Cellulase pretreatment	5	37
iv.	Simultaneous laccase and cellulase pretreatment	3	30
v.	Simultaneous laccase and cellulase pretreatment	3	37
vi.	Simultaneous laccase and cellulase pretreatment	5	30
vii.	Simultaneous laccase and cellulase pretreatment	5	37

### 2.8 Simultaneous saccharification and fermentation

After investigating the conditions for pretreatment, the biomasses of DB, HM, SP, PA, and PK were subjected to simultaneous saccharification and fermentation. The biomass (0.25 g) was inoculated with 10% (v/v) inoculum of MH 10 in a buffer of pH 3 and kept at 50°C for 24 h, as determined in previous experiments. The biomasses were added with either laccase only or with laccase and cellulase along with the strain MH 10. After incubation, pectinase was obtained via centrifugation and CFCS was used as the crude pectinase preparation.

### 2.9 Pectinase assay

Pectin (0.5% w/v) prepared in 50 mM Tris HCl (pH 8.5) was used as a substrate. Pectinase preparation from MH 1 (25 µL) was mixed with 25 µL of the substrate and incubated for 10 min at 50°C (3). The amount of reducing sugars released by the enzyme was determined by dinitrosalicylic acid method [26] and IU/mL of the pectinase was calculated using the standard curve prepared from different concentrations of D- galacturonic acid (S1 Fig). One international unit of pectinase activity was defined as the amount of enzyme required to liberate 1 µmole of D-galacturonic acid per mL under the assay conditions [27].

### 2.10 Gravimetric analysis of plant biomass

Water soluble fraction, ethanol soluble fraction, hemicellulose, lignin and cellulosic content of plant biomass samples were determined gravimetrically [28]. Briefly, 75 mL of distilled water was added to the sample (1 g) and boiled for 1 h. The slurry was filtered, and the same procedure was repeated. After filtering, the residue was rinsed with cold water and dried at 60°C. The dried sample was weighed and weight loss was calculated as water soluble fraction.

The ethanol-soluble fraction was evaluated by adding ethanol (100 mL) to residues and boiled for 2 h. The sample was then filtered and rinsed twice with distilled water. The sample was then dried at 60°C and weighed again. The fraction loss was taken as the ethanol-soluble component.


Pectin content = Initial weight – Remaining weight after above treatment

To determine lignin content, residues from the preceding phase were treated with 5 mL of 12% (w/v) sodium hypochlorite, 2 mL of 10% (v/v) acetic acid, and 25 mL distilled water. The reaction mixture was heated at 70°C for 1 h. The residues were then filtered and washed five times with distilled, two times with acetone, and once with ether, and then dried at 60°C for 24 h.

For hemicellulose content, the residue from the previous step was mixed with 24% KOH (20 mL) and kept at 20°C for 2 h. The mixture was filtered and washed with 5% acetic acid (v/v), followed by water, acetone, and ether. The remaining components were dried overnight at 60°C. To determine the hemicellulose content, the following equation was used:


Hemicellulose content = Recovered residue after lignin content treatment − Residue remaining after above treatment

Residues remain from the hemi cellulosic content were taken as the cellulose component.

### 2.11 Extraction of pectin from halophytic plant

The simultaneous saccharification and fermentation of biomass from *P. antidotale* provided promising results, however, the pectin content of this biomass has not been reported earlier. Therefore, the pectin content of this plant was determined. Orange peel (OP) was used as a positive control for pectin extraction. Pectin extraction was performed by following a protocol reported by Kratchanova et al. [29] with slight modifications. Briefly, 1 g of plant biomass was added to 50 mL deionized water and pH was adjusted to 1.5 using 0.5 M HCl. It was then stirred at 80ºC for 1 h; the contents were filtered and filtrate was cooled down. Afterwards, 96% (v/v) ethanol was added to filtrate in equal ratio and kept for 1 h at ambient temperature. The contents were filtered and residues were washed with 70% (v/v) acidic ethanol and then with 70% (v/v) until the pH of the pectin gel became neutral. The pectin gel was washed with 96% (v/v) ethanol and then dried at 60ºC for 24 h and weighed.

### 2.12 Scanning electron microscopy

Native (untreated), laccase-treated, and laccase-treated along with MH 10 fermented samples of OP and PA were examined via Scanning Electron microscope to investigate the structural changes. The sample was coated with 250 A° gold layer using an ion sputtering device (JEC-1500, JEOL USA) and scanned at magnifications of 700x, 1000x and 1200x at a voltage of 10 kV.

### 2.13 Fourier transform infrared spectroscopy

Native, laccase-treated and laccase-treated along with MH 10 fermented samples of OP and PA were oven dried, mixed with KBr at a ratio of 1:200 and then vacuum pressed into pellets. The samples were subjected to a Fourier Transform Infrared Spectroscopy (FTIR) on JASCO FTIR-4200 between wavelengths of 4000 and 500 cm^-1^.

### 2.14 Statistical analysis

The data was analyzed by the Origin pro 18 software and values of mean with standard deviation are mention. The graphical illustration has been made using the same software.

## 3. Result

### 3.1 Saccharification of halophytic plant biomass

**Fig 1:**
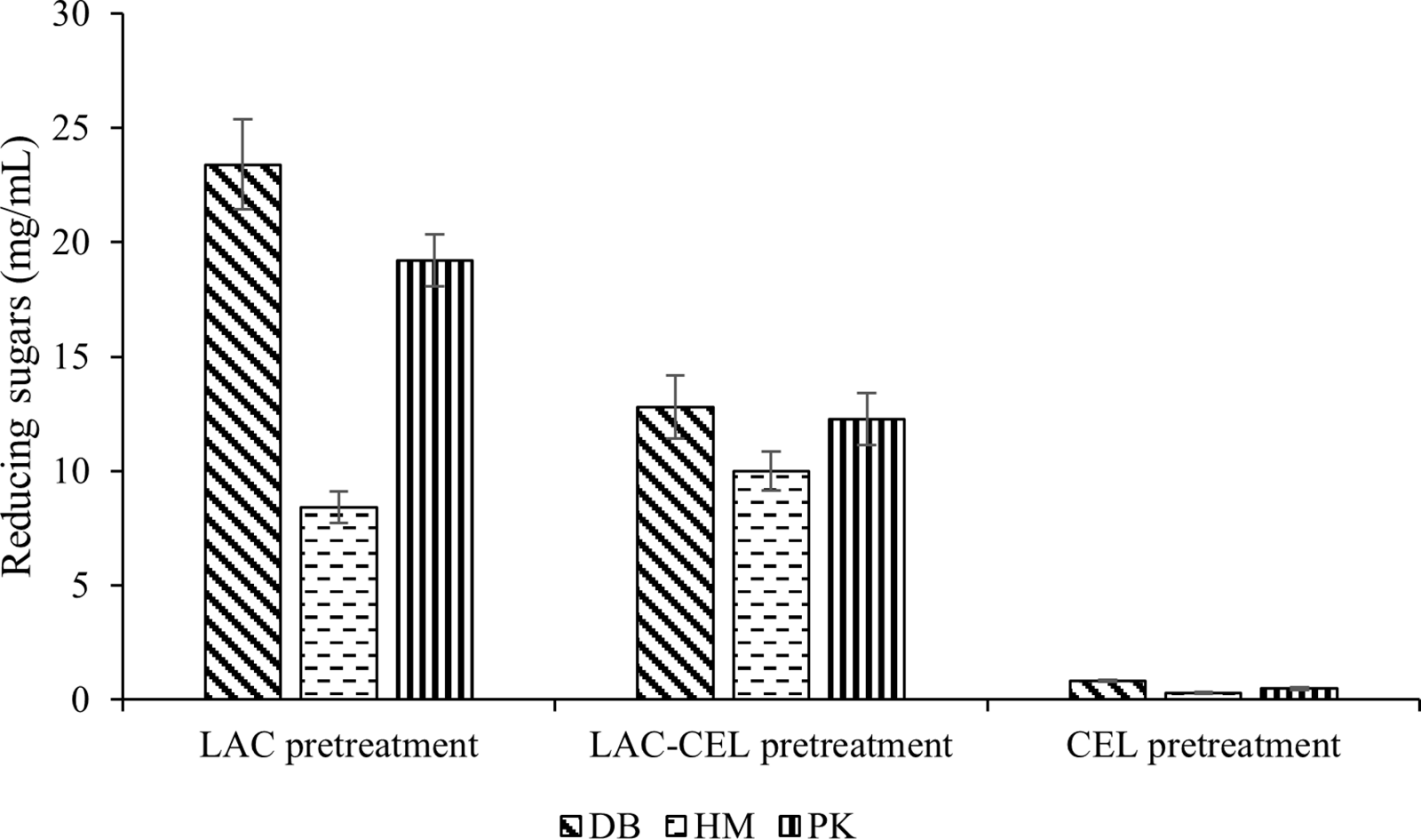
Enzymatic Saccharification of *Desmostachya bipinnata* (DB), *Halopyrum mucronatum* (HM) and *Phragmites*s *karka* (PK) in different combinations, i.e., laccase pretreatment (*LAC* pretreatment), laccase-cellulase pretreatment (*LAC-CEL* pretreatment) and cellulase pretreatment (*CEL* pretreatment).

In this study, laccase from *T. pubescens* MB 89 was applied for pretreatment along with cellulase from MH 1 strain for the saccharification of biomass from halophytes. The data showed that the laccase pretreatment of biomass from the halophytic plants enhanced reducing sugar production when compared to cellulase pretreatment alone (Fig 1). Indeed, cellulase alone proved to be ineffective as a saccharifying agent indicating the importance of lignin removal. Laccase alone was found to be a better pretreatment agent for biomass from DB and PK.

### 3.2 Impact of temperature and pH on saccharification

**Fig 2:**
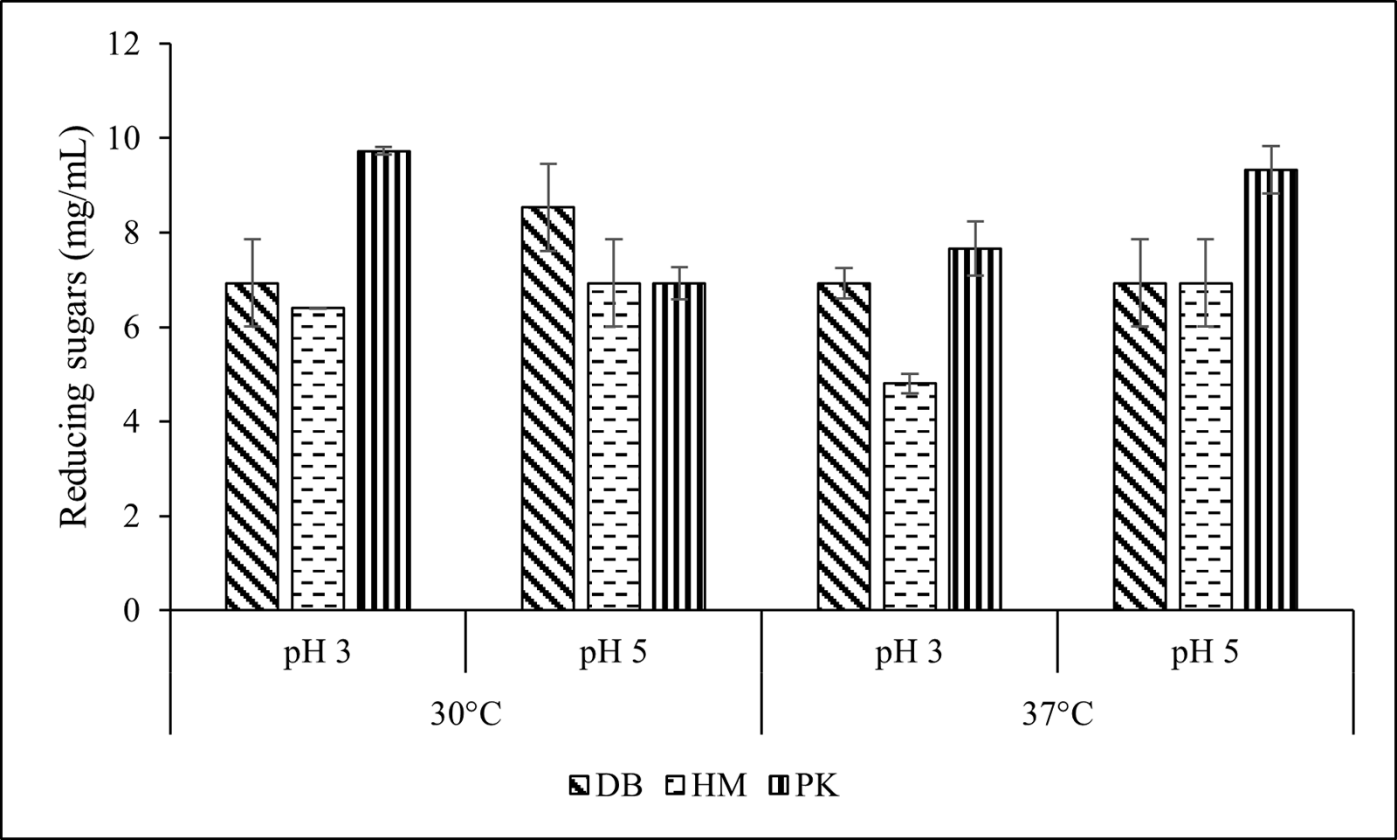
Saccharification of *Desmostachya bipinnata* (DB), *Halopyrum mucronatum* (HM) and *Phragmites karka* (PK) with combined pretreatment of laccase and cellulase at pH 3 and 5 incubated at 30 and 37°C.

Since the common optimum conditions for fungal laccase and bacterial cellulase were difficult to adjust, therefore, the saccharification was investigated at two previously determined optimum temperatures and pH values for laccase and cellulase. The data showed that saccharification of PK at 30^o^C and pH 3 yielded the highest amount of reducing sugars of 9.8 mg mL^-1^ (Fig 2). For DB, saccharification improved when the reaction was incubated at 30^o^C in pH 5 where it 8.7 mg mL^-1^ of reducing sugars were obtained. However, the yields of reducing sugars did not vary greatly (4.9–6.2 mg mL^-1^) across the experimental conditions when the HM biomass was saccharified.

### 3.3 Simultaneous enzyme mediated pretreatment of biomass and pectinase production by *B. vallismortis* MH 10

Except for PA, the pectinase titers were increased by several-folds when the biomasses from halophytic plants were added with laccase and cellulase along with the MH 10 strain (Table 2). Indeed, the titers obtained using wild biomass exceeded 10 IU mL^-1^ than those obtained in commercial pectin-containing medium (4.78 IU mL^-1^). Interestingly, cultivation of MH 10 in the presence of PA biomass with laccase only yielded 17.39 IU mL^-1^ pectinase, which increased to 21.83 IU mL^-1^ when cellulase was also added to the medium. However, the expenses on the cultivation and incubation time for cellulase production cannot justify this ~20% increase in the pectinase titers. Thus, further analysis was conducted using PA plant biomass supplemented with laccase only.

**Table 2:** Pectinase production by *B. vallismortis* MH 10 in presence of plant biomass *Desmostachya bipinnata* (DB), *Halopyrum mucronatum* (HM), *Salvadora persica* (SP)*, Panicum antidotale* (PA) and *Phragmites*s *karka* (PK) in presence of laccase and cellulase.

	Pectinase production (IU/mL) by the strain MH 10		
Biomass	Without lacasse and cellulase supplementation	With supplementation of laccase	With supplementation of laccase and cellulase
DB	1.5	1.23 ± 0.21	16.28
HM	2.55 ± 0.30	3	17.14 ± 0.42
PK	1.54 ± 0.10	1.92 ± 0.10	10.64 ± 0.39
SP	2.02 ± 0.18	2.42 ± 0.42	12.25 ± 1.15
PA	5.07 ± 0.56	17.39 ± 1.28	21.83 ± 1.33
Commercial Pectin	4.78 ± 0.49	ND	ND

*ND = not determined (Commercially available pectin does not need enzyme treatment)

### 3.4 Time-dependent release of reducing sugars from *P. antidotale* biomass

Studies on the release of reducing sugars with respect to time indicated laccase-mediated sugar release by 226 mg g^-1^ from PA biomass in the medium lacking MH 10 inoculation (Table 3) which did not exceed 626 mg g^-1^ even after 24 h of incubation. The amount of reducing sugar was found to be only 200 mg g^-1^ in the flask containing MH 10 which remained low until 4 h, affirming bacterial growth by utilizing laccase-driven reducing sugars. It was perceived that after initial adjustment or the lag phase, the organism derived reducing sugars from the biomass by itself and hence, the highest reducing sugar amount was obtained at 24 h (873 mg g^-1^). Nevertheless, laccase pretreatment significantly enhanced the release of reducing sugars in comparison to the untreated substrate.

**Table 3:** Estimation of reducing sugar with respect to time interval in untreated (PA), laccase pretreated (PA) and laccase-MH 10 pretreated (PA).

Saccharification	Release of reducing sugars (mg/g) by MH 10		Release of reducing sugars by Laccase pretreatment of PA biomass (mg g^-1^)
Time (min)	Untreated PA biomass	Laccase and MH 10 treated PA biomass	
30	0	200	226
90	400	520	520
240	586	560	626
1440	560	873	626

### 3.5 Time-dependent laccase activity in production medium

This study aimed to investigate the enzyme activity and stability of supplemented laccase, as well as the potential of the MH 10 strain to produce laccase or other lignin-degrading enzymes. Therefore, to exclude the role of laccase produced by strain MH 10 of *B. vallismortis*, any residual laccase activity was also assayed. The results in Table 4 indicated that MH 10 produced low levels of an unstable laccase enzyme, whereas, the supplemented laccase from *T. pubescens* MB 89 remained active for up to 90 min before losing its activity.

**Table 4:** Estimation of laccase activity with respect to time using *Panicum antidotale* biomass.

Time (min)	Laccase activity (IU/mL) in the flasks inoculated with MH 10		MB 89 Laccase pretreated PA biomass (IU/mL)
	Untreated PA biomass	Laccase treated PA biomass	
30	0	15.6 ± 2.07	117.53 ± 10.63
90	233.5 ± 14.8	66.66 ± 4.61	9.6 ± 4.15
240	280.8 ± 10.18	234 ± 5.09	0
1440	93.6 ± 10.18	216	0

### 3.6 Time-dependent pectinase production

The production of pectinase by MH 10 was monitored over time. Results indicated that pectinase production was commenced within 1.5 h of the bacterial cultivation and reached to its peak level in 24 h. The pectinase titers remained higher in the medium supplemented with laccase and the maximum amount (19.17 IU mL ⁻ ¹) was obtained after 24 h (Table 5).

**Table 5:** Pectinase production kinetics by MH 10 in untreated and laccase-pretreated PA biomass.

Time (min)	Pectinase production (IU mL^-1^) by the strain MH 10 in the medium containing		Laccase pretreated PA biomass (Without MH 10)
	Untreated PA biomass	Laccase treated PA biomass	
30	0	0	0
90	5.43 ± 0.11	5.54 ± 0.12	0
240	6.3 ± 0.43	8.86 ± 0.81	0
1440	5.83 ± 0.66	19.17 ± 0.75	0

### 3.7 Gravimetric analysis and pectin content determination in *Panicum antidotale* biomass

The chemical compositions of untreated, laccase-pretreated, and laccase-supplemented fermented residues of OP and PA were examined (Table 6). It was found that untreated OP contained a lower lignin content (3.2%) that was similar to its reported composition. The amount of lignin and hemicellulose in the PA biomass was higher than OP. However, the cellulosic content increased to 57.9% (Table 6) following pretreatment, indicating a reduction in other constituents. The lignin content in OP decreased from 3.2% to 0.77% in laccase-treated and to 0.21% in laccase-treated and fermented OP biomass. Likewise, the lignin content in PA decreased from 6.9% in native biomass to 4.3% and 3.6% in laccase-treated and fermented substrates, respectively. These results confirmed the impact of laccase on both substrates, *i.e.,* OP and PA.

**Table 6:** Fractionation of native (untreated), laccase (LAC-) pretreated, laccase pretreated and fermented (Lac + MH10) orange Peels and *Panicum antidotale* biomass into lignin, hemicellulose and cellulose.

Fraction	Percentage of dry weight of fraction in					
	Orange peels			*Panicum antidotale* biomass		
	Untreated	*LAC*- pretreated	*LAC* + MH10	Untreated	*LAC*- pretreated	*LAC* + MH10
Water soluble fraction	64.2	19.67	5.52	4.5	1.3	5.01
Ethanol soluble fraction	0.4	2.08	5.78	5.2	14.4	6.29
Lignin	3.2	0.77	0.21	6.9	4.3	3.6
Hemicellulose	2.9	14.04	0.49	25.5	25.8	43.43
Cellulose	29.2	63.4	88.1	57.9	53.3	41.6

The removal of lignin during bacterial fermentation led to an increase in the percentages of hemicellulose and cellulose. Interestingly, the cellulose content of PA biomass decreased after bacterial fermentation, suggesting that MH 10 strain has a stronger affinity for the halophytic substrate than the control.

Pectin content in *P. antidotale* biomass has not been previously reported; therefore, it was investigated in this study (Table 7). Pectin was extracted from OP to validate the method. The results showed that PA biomass contained 2.8% pectin and MH 10 utilized 94.2% of this pectin content for pectinase production (Table 7). Although, the amount of pectin in OP was higher (15%) yet MH 10 utilized lower amount of this pectin inferring preference of this organism for halophytic biomass. MH 10 produced 25 IU mL^-1^ of pectinase when PA biomass was utilized as a substrate with 315.6 IU g^-1^ of pectin from PA and 76.32 IU g^-1^ pectin from OP (Table 7).

**Table 7:** Pectin content in native (untreated), laccase (LAC-) pretreated, laccase pretreated and fermented (Lac + MH10) Orange peels and *Panicum antidotale* biomass. The substrates were used for the production of pectinase and the pectinase titers presented.

Substrate	Treatment	Initial biomass (g)	After drying/treatment (g)	Remaining pectin content (%)	Pectinase (IU mL^-1^)	Pectinase produced/gram of biomass (IU g^-1^)	Total Pectinase/gram of pectin used (IU g^-1^)
Orange peels	Untreated	1	–	14.9	ND	ND	ND
	Lac-pretreated	5	3.4	7.5	ND	ND	ND
	(*LAC* + MH 10)	5	2.8	2.03	49.08	981.6	76.32
PA biomass	Untreated	3	2.5	2.8	ND	ND	ND
	Lac- pretreated	3	2.5	0.24	ND	ND	ND
	(*LAC* + MH 10)	3	2.5	0.16	25	833.3	315.65

*ND=not determined

### 3.8 Structural analysis of the *Panicum antidotale* and orange peel plant biomass

**Fig 3:**
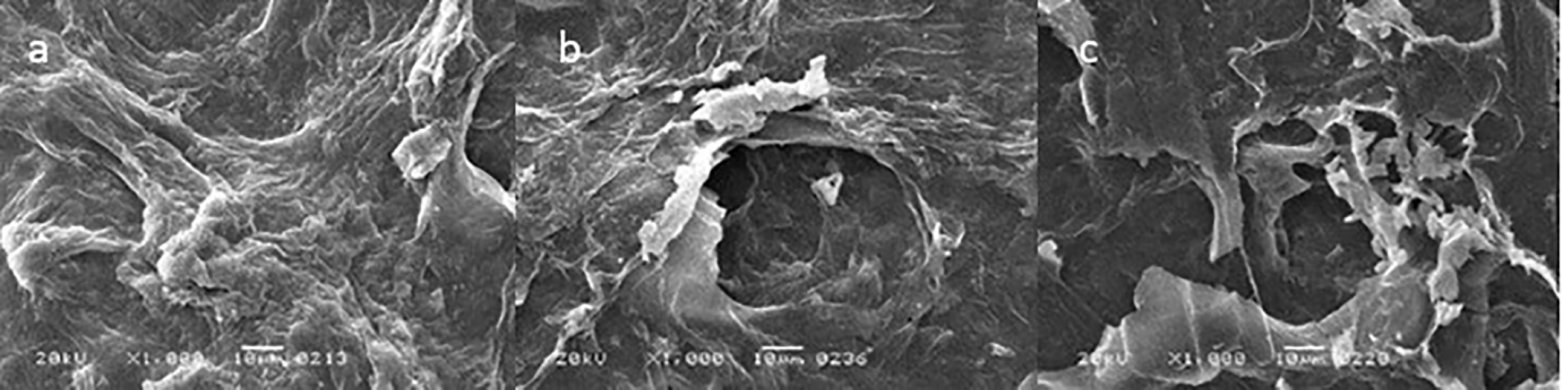
Surface images of (A) untreated (B) laccase-treated (C) and fermented laccase-treated peels by scanning electron microscopy.

**Fig 4:**
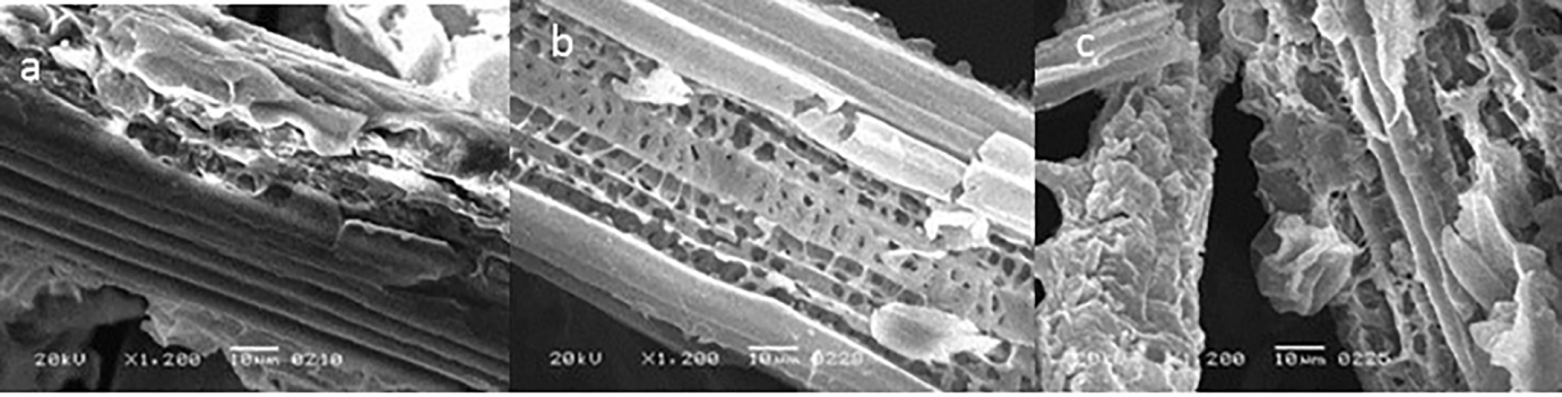
Surface images of (A) untreated (B) laccase-treated (C) and fermented laccase-treated *P. antidotale* by scanning electron microscopy.

The surface structures of the OP and PA biomass were investigated using Scanning Electron microscope. Untreated samples showed an intact and compact structure (Figs 3a and 4a). However, laccase pretreatment caused deformities in the OP (Fig 3b) and formed pores in the PA biomass (Fig 4b). Moreover, bacterial fermentation led to further degradation of both OP and PA biomass owing to the action of pectinase (Figs 3c,4c).

**Fig 5:**
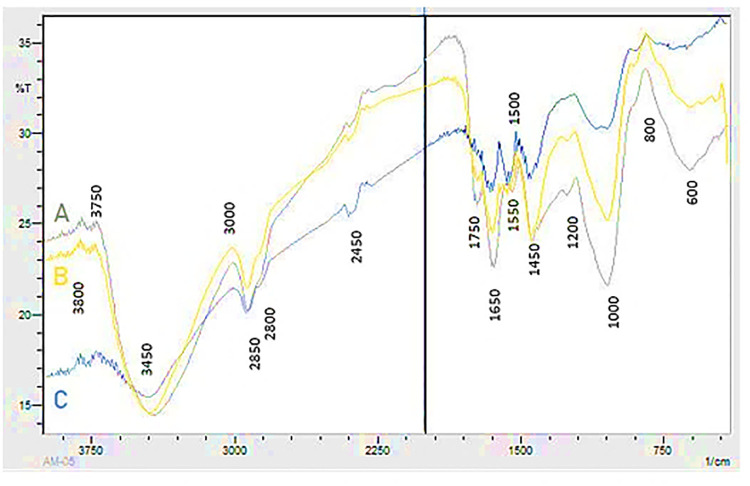
FTIR of (A) untreated orange peels (B) laccase treated orange peels and (C) orange peels showing simultaneous treatment of laccase and pectinase production.

**Fig 6:**
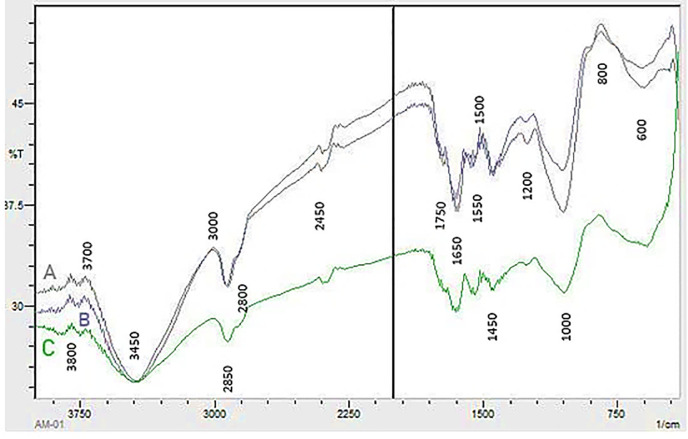
FTIR of (A) untreated *P. antidotale* biomass (B) laccase treated *P. antidotale*biomass (C) *P. antidotale* biomass showing simultaneous treatment of laccase and pectinase production.

FTIR analysis of OP (Fig 5; S1 Table) and PA biomass (Fig 6; S2 Table) demonstrated the effects of laccase pretreatment and bacterial fermentation on plant biomass composition compared to untreated samples. Lignin peaks were mainly observed in the region of 1200–1600 cm^-1^ in native samples of PA and OP, more specifically in 1510–1250 cm^-1^ region. Whereas, peaks around 3450 cm^-1^, 1650 cm^-1^ and 1250 cm^-1^ showed OH stretching of lignin, C = O stretching vibration in conjugated carbonyl of lignin and C-O vibration related to G lignin, respectively. Laccase pretreatment led to delignification in both biomasses of PA and OP that was evident by changes in the intensities of the peaks related to lignin. The peaks around 2900 cm^-1^ were due to –OH vibrations which is usually found in the pectic acid components of the biomass. The esterified carboxyl group of the pectin structure is linked to the band at approximately 1750 cm^-1^. The changes in intensities in the region between 2900 and 1650 cm^-1^ presented pectin degradation by pectinase in our fermented biomass sample.

## 4. Discussion

Lignocellulose biomass is reported to be recalcitrant and requires pretreatment for efficient consumption of its components by microorganism. Among the biological pretreatment methods, lignin-degrading enzymes, such as laccases, are environmentally friendly and effective for lignin removal. Earlier studies have reported an improvement in saccharification and pectin extraction from plant biomass after treatment with laccase [21,30]. Pretreatment methods usually increase the yield of reducing sugars, as reported earlier for the alkaline pretreatment of corncob, where the total reducing sugar yield increased by 33.6% [5]. Earlier studies on simultaneous pretreatment and saccharification of sugarcane with laccase and cellulase yielded 1.15% reducing sugars [24]. In this study, laccase pretreatment caused the release of more reducing sugars than the untreated or cellulase pretreated biomasses. Nonetheless, the biomass from PA presented a sustainable chemical feedstock. Environment friendly approaches, such as, enzymatic treatment, can render the processes align with the sustainable development goals suggested by the United Nations.

Simultaneous pretreatment and saccharification have been shown to be an efficient and cost-effective approach [31]. Hafeez et al. [24] found that enzymatic pretreatment and saccharification of sugarcane bagasse along with fermentation is an effective strategy for value-added product synthesis. Since, enzyme activity and stability depend on pH [14] and temperature [24,32], therefore, different temperatures and pH for simultaneous saccharification and fermentation were determined in this study. The impact of temperature and pH was variable when different biomasses were saccharified. It was found that pH 3 provided better condition for the saccharification of DB and PK indicating more pronounced effect of laccase than the cellulase. It is worth noting that the pH 3 was reported as an optimum pH for the laccase activity by *T. pubsescens* MB 89 [23].

Considering our initial experiments on PA biomass, it was also included in this study. The strain produced the highest titers of reducing sugars by saccharifying PA biomass in 24 h that corroborated with the maximum growth of this strain at 26 h [13]. Indeed, the effectiveness of pretreatment observed in this study was better than that reported earlier where 3.3 mg g ⁻ ¹ of reducing sugars by acid-treated PA and 40 mg g ⁻ ¹ by PK were obtained [7].

Any effect of laccase produced by the fermenting bacterium, *B. vallismortis* was also excluded in this study. This was in the light of an earlier report on laccase production by the *B. vallismortis* fmb 103 strain [32]. The data indicated about low titers of thermally unstable laccase produced by the strain MH 10 that did not contribute in pretreatment of the substrate and hence, the fungal laccase pretreatment was an essentially required. An increase in pectinase titers in the presence of laccase pretreated PA biomass was aligned with studies where the addition of ligninolytic fungal species as a pretreatment agent enhanced digestion by 90% of switchgrass biomass [33].

The pectin content of PA has not been reported earlier. Indeed, very few studies describe pectin content in halophyte derived biomasses. Therefore, pectin and other contents in PA biomass were determined. To affirm the findings, OP biomass was utilized for the comparative analysis. It was found that untreated OP contained a lower lignin content (3.2%), consistent with a previous report [34]. Similarly, the lignin and hemicellulose contents of untreated PA biomass affirmed the earlier findings by Ansari et al. [7]. The removal of lignin during bacterial fermentation led to an increase in the percentages of hemicellulose and cellulose. Interestingly, the cellulose content of PA biomass decreased after bacterial fermentation with the MH 10 strain, suggesting that MH 10 has a stronger affinity for the halophytic substrate than the OP control. The pectin content in PA biomass was much lower yet the strain MH 10 produced 25 IU mL^-1^ of pectinase utilizing this biomass which was comparable to the 20 IU mL^-1^ pectinase produced by the same strain in the presence of another halophyte, *Cressia cretica*, that contained a higher content of pectin (~17%) [14]. The higher enzyme yield from *P. antidotale* biomass compared to OP may be attributed to differences in substrate composition and structural complexity as explained by Hassan et al. [14]. The findings suggest that substrate composition remarkably influence the microbial metabolite synthesis [34,35].

The effect of pretreatment and bacterial fermentation was visualized by SEM and FTIR analysis. The laccase pretreatment caused deformities in the OP and formed pores in the PA biomass which was previously reported for pomelo peels by Li et al. [29]. The structure became extremely disordered after fermentation. These observations are consistent with the findings of Kucharska et al. [36].

The FTIR spectra of untreated, MH10-treated, and laccase+MH10 treated samples were analyzed to identify structural and chemical modifications in the lignocellulosic biomass. The characteristic absorption bands corresponding to the major functional groups of lignin and pectin were observed in the native samples, with notable variations in their relative intensities after lignin degradation and pectin fermentation. Particularly, the IR spectra recorded in the 500–4000 cm ⁻ ¹ range showed that the untreated OP sample aligned with the findings of Orozco et al. [37]. Moreover, the spectrum for untreated PA resembled that of *Panicum maximum* [38], indicating a similar biomass composition within the genus.

The pectinase produced from Bacillus valismortis MH10 could be used for variety of applications such as fruit juice treatment [39], detox juice clarification and yield enhancement [40], oil extraction, degumming of jute fibers, tea and coffee fermentation [41,42] etc.

## 5. Conclusion

Pectinase production from halophytic biomass is a novel and sustainable approach, as they are non-conventional resource which is under exploration. The Biomass of *Panicum antidotale* was used for the first time for pectinase production. Simultaneous biological pretreatment and bacterial fermentation appeared as a promising strategy for pectinase production. Although biomass of *P. antidotale* contained meager quantity of pectin, but with the laccase-mediated pretreatment, it became available to *B. vallismortis* MH 10 which produces 315 IU pectinase per g of pectin. The findings suggest that the strain has more affinity towards halophytic substrate utilizing 94% of its pectin but very low quantity of pectin present in orange peels was consumed. Laccase employment caused removal of lignin from plant biomass which was confirmed by the SEM and FTIR. Further research is needed to explore the utilization of PA biomass completely with the production of value-added products in addition to pectinase.

## Supporting information

S1 Fig Standard curve of galacturonic acid (pH 4.8). (DOCX)

S1 Table Main absorption bands in orange peels biomass. (DOCX)

S2 Table Main absorption bands in *P. antidotale* biomass. (DOCX)
