# Supplementary material for: Biological pretreatment and fermentation of Panicum antidotale biomass for pectinase production by Bacillus vallismortis
Source: PLoS One. 2026 Jan 23;21(1):e0339181. doi: 10.1371/journal.pone.0339181 (PMC12829774; doi:10.1371/journal.pone.0339181)
Supplement: S1 Fig — (DOCX) [file pone.0339181.s001.docx]

**Biological Pretreatment and Fermentation of *Panicum antidotale* Biomass for Pectinase Production by *Bacillus vallismortis***

Amal Siraj^a,b^, Uroosa Ejaz^c^, Masooma Hassan^a^, Mohammed Alorabi^d^, Abdullah K. Alanazi^e^, Muhammad Sohail^a*^

^a^Department of Microbiology, University of Karachi, Karachi 75270, Pakistan

^b^Department of Applied Sciences, Hamdard University, Karachi-74600, Pakistan

^c^Department of Biosciences, Faculty of Life Science, SZABIST University, Karachi 75600, Pakistan

^d^Department of Biotechnology, College of Sciences, Taif University, 21944 Taif, Saudi Arabia

^e^Department of Chemistry, College of Science, Taif University, 21944 Taif, Saudi Arabia

*Author for all correspondence: [msohail@uok.edu.pk](mailto:msohail@uok.edu.pk) ORCiD: 0000-0002-7208-9441


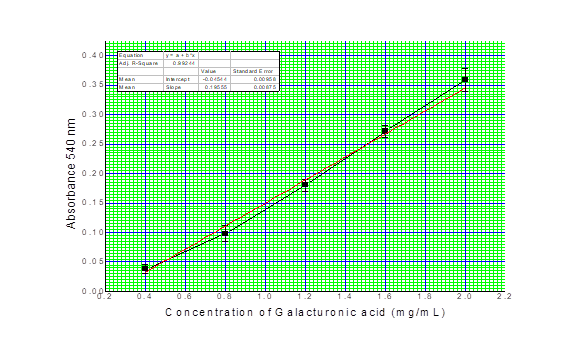


**Fig. S1** Standard curve of galacturonic acid (pH 4.8).
