# Supplementary material for: Biological pretreatment and fermentation of Panicum antidotale biomass for pectinase production by Bacillus vallismortis
Source: PLoS One. 2026 Jan 23;21(1):e0339181. doi: 10.1371/journal.pone.0339181 (PMC12829774; doi:10.1371/journal.pone.0339181)
Supplement: S1 Table — (DOCX) [file pone.0339181.s002.docx]

**Biological Pretreatment and Fermentation of *Panicum antidotale* Biomass for Pectinase Production by *Bacillus vallismortis***

Amal Siraj^a,b^, Uroosa Ejaz^c^, Masooma Hassan^a^, Mohammed Alorabi^d^, Abdullah K. Alanazi^e^, Muhammad Sohail^a*^

^a^Department of Microbiology, University of Karachi, Karachi 75270, Pakistan

^b^Department of Applied Sciences, Hamdard University, Karachi-74600, Pakistan

^c^Department of Biosciences, Faculty of Life Science, SZABIST University, Karachi 75600, Pakistan

^d^Department of Biotechnology, College of Sciences, Taif University, 21944 Taif, Saudi Arabia

^e^Department of Chemistry, College of Science, Taif University, 21944 Taif, Saudi Arabia

*Author for all correspondence: [msohail@uok.edu.pk](mailto:msohail@uok.edu.pk) ORCiD: 0000-0002-7208-9441

**Table S1**. Main absorption bands in orange peels biomass.

| **Vibration** | **Peak** | **Untreated orange peels** | **Laccase treated orange peels** | **Simultaneous treatment of laccase and pectinase production using orange peels** |
| --- | --- | --- | --- | --- |
| OH stretching | 3450 | Broad and strong | Broad and strong | Broad and weaker |
| CH stretching | 2900 | Stronger, medium | Stronger, medium | Stronger, medium |
| CH stretching | 2850 | Weakest | Weaker | Weak |
| Weak peaks may appear in this region due to different types of bonds and it is not a fingerprint region | 2450 | Weakest | Weakest | Small peak |
| C=O stretching | 1750 | Small peak | No peak | No peak |
| C=C stretching | 1650 | Strong sharp | Strong sharp | Weak |
| CH2 and CH3 vibrations | 1450 | Strong sharp | Strong sharp | Weak |
| COH & COR vibrations | 1000 | Strong broad | Strong broad | Weak broad |
| Aromatic CH binding of lignin | 600 | Broader peak | Broader peak | No peak |
