# Supplementary material for: Biological pretreatment and fermentation of Panicum antidotale biomass for pectinase production by Bacillus vallismortis
Source: PLoS One. 2026 Jan 23;21(1):e0339181. doi: 10.1371/journal.pone.0339181 (PMC12829774; doi:10.1371/journal.pone.0339181)
Supplement: S2 Table — (DOCX) [file pone.0339181.s003.docx]

**Biological Pretreatment and Fermentation of *Panicum antidotale* Biomass for Pectinase Production by *Bacillus vallismortis***

Amal Siraj^a,b^, Uroosa Ejaz^c^, Masooma Hassan^a^, Mohammed Alorabi^d^, Abdullah K. Alanazi^e^, Muhammad Sohail^a*^

^a^Department of Microbiology, University of Karachi, Karachi 75270, Pakistan

^b^Department of Applied Sciences, Hamdard University, Karachi-74600, Pakistan

^c^Department of Biosciences, Faculty of Life Science, SZABIST University, Karachi 75600, Pakistan

^d^Department of Biotechnology, College of Sciences, Taif University, 21944 Taif, Saudi Arabia

^e^Department of Chemistry, College of Science, Taif University, 21944 Taif, Saudi Arabia

*Author for all correspondence: [msohail@uok.edu.pk](mailto:msohail@uok.edu.pk) ORCiD: 0000-0002-7208-9441

**Table S2**. Main absorption bands in *P. antidotale* biomass.

| **Vibration** | **Peak** | **Untreated *P. antidotale*** | **Laccase treated *P. antidotale*** | **Simultaneous treatment of laccase and pectinase production using** ***P. antidotale*** |
| --- | --- | --- | --- | --- |
| OH stretching | 3450 | Broad and strong | Broad and strong | Broad and weaker |
| CH stretching | 2900 | Stronger, medium | Stronger, medium | Stronger, medium |
| CH stretching | 2850 | Weakest | Weaker | Weak |
| C=C stretching | 1650 | Medium sharp | Medium sharp | Weak |
| CH2 and CH3 vibrations | 1450 | Weak | Weak | Too weak |
| COH & COR vibrations | 1000 | Strong broad | Strong broad | Weak Broad |
| Aromatic CH binding of lignin | 600 | Too weak | Too weak | Broad and weak |
